# Autocrine Netrin Function Inhibits Glioma Cell Motility and Promotes Focal Adhesion Formation

**DOI:** 10.1371/journal.pone.0025408

**Published:** 2011-09-28

**Authors:** Andrew A. Jarjour, Margaret Durko, Tamarah L. Luk, Nathalie Marçal, Masoud Shekarabi, Timothy E. Kennedy

**Affiliations:** Department of Neurology and Neurosurgery, Montreal Neurological Institute, McGill University, Montreal, Quebec, Canada; Yale University School of Medicine, United States of America

## Abstract

Deregulation of mechanisms that control cell motility plays a key role in tumor progression by promoting tumor cell dissemination. Secreted netrins and their receptors, Deleted in Colorectal Cancer (DCC), neogenin, and the UNC5 homologues, regulate cell and axon migration, cell adhesion, and tissue morphogenesis. Netrin and netrin receptor expression have previously been shown to be disrupted in invasive tumors, including glioblastoma. We determined that the human glioblastoma cell lines U87, U343, and U373 all express neogenin, UNC5 homologues, and netrin-1 or netrin-3, but only U87 cells express DCC. Using transfilter migration assays, we demonstrate DCC-dependent chemoattractant migration of U87 cells up a gradient of netrin-1. In contrast, U343 and U373 cells, which do not express DCC, were neither attracted nor repelled. Ectopic expression of DCC by U343 and U373 cells resulted in these cells becoming competent to respond to a gradient of netrin-1 as a chemoattractant, and also slowed their rate of spontaneous migration. Here, in addition to netrins' well-characterized chemotropic activity, we demonstrate an autocrine function for netrin-1 and netrin-3 in U87 and U373 cells that slows migration. We provide evidence that netrins promote the maturation of focal complexes, structures associated with cell movement, into focal adhesions. Consistent with this, netrin, DCC, and UNC5 homologues were associated with focal adhesions, but not focal complexes. Disrupting netrin or DCC function did not alter cell proliferation or survival. Our findings provide evidence that DCC can slow cell migration, and that neogenin and UNC5 homologues are not sufficient to substitute for DCC function in these cells. Furthermore, we identify a role for netrins as autocrine inhibitors of cell motility that promote focal adhesion formation. These findings suggest that disruption of netrin signalling may disable a mechanism that normally restrains inappropriate cell migration.

## Introduction

Cell migration is essential for normal embryonic development, wound healing, and immunity but can be devastating in tumor invasion and metastasis. Netrins are secreted, laminin-related proteins that direct cell and axon migration during neural development (reviewed by [1]). Netrin-1 and netrin receptors DCC, the DCC paralogue neogenin, and UNC5 proteins, are also expressed in many adult tissues [2]–[9], but their function in mature tissues is poorly understood. Netrin-1 is widely expressed by neurons and glia in the adult CNS [5] [10]. Reduced expression of netrin-1 has been documented in brain tumors, including glioblastoma [4], however, a role for netrins regulating brain tumor cell migration has not been established.

Although substantial evidence suggests an anti-oncogenic role for DCC, how disruption of netrin signaling might contribute to malignancy is poorly understood. In colorectal cancer, allelic deletion involving chromosome 18q21 occurs in >70% of tumors [11] and the *dcc* gene was first identified as a putative tumor suppressor from this chromosomal deletion [2]. *Dcc* expression is reduced in many cancers, including most high-grade gliomas [12] [13]and loss of DCC correlates with the development of highly invasive glioblastoma multiformae [13]. Furthermore, ectopic expression of *dcc* in transformed epithelial cells reduced tumorigenicity [14] [15], and expression of DCC antisense RNA in transformed fibroblasts resulted in an increased growth rate, anchorage independence, and tumorigenicity when the cells were transplanted into nude mice [16]. No increased incidence of tumor formation has been detected in conventional DCC knockout mice [17], however, conclusions drawn from this study were complicated by the possibility that tumors may not have had time to develop due to the early post-natal lethality of DCC knockouts. Unc5 homologue netrin receptors signal chemorepulsion, and co-expression of DCC often facilitates UNC5 function (reviewed by [1]). Four UNC5s, UNC5A-D, are expressed in mammals. Altered expression of UNC5A, B, C, and D has been detected in various cancers and tumor cell lines [6], [18] [19].

Here we investigated the possibility that netrins and netrin receptors influence tumor cell migration. Using human glioblastoma cell lines, we provide evidence that DCC is required for chemoattraction to netrin-1 and slows the rate of spontaneous cell migration. Our findings support a role for netrins as autocrine inhibitors of cell motility that regulate focal adhesions (FA).

## Results

### Glioblastoma cells express netrin and netrin receptors

**Figure 1:**
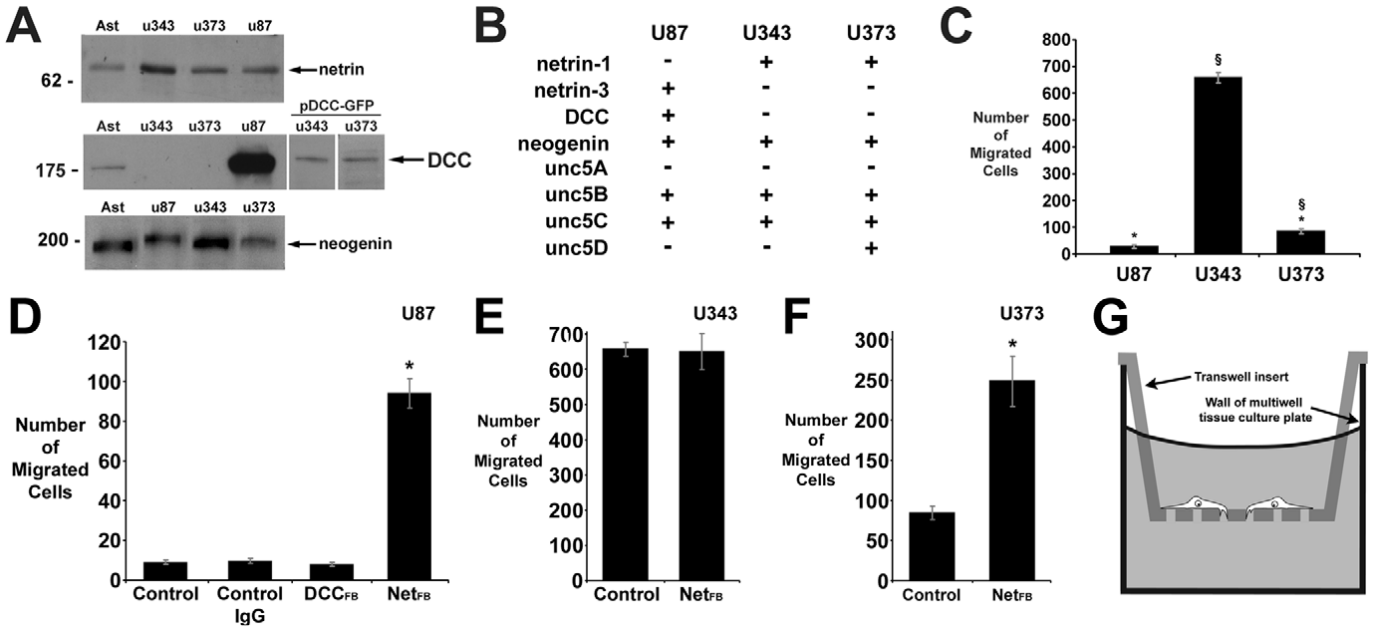
Glioblastoma cell lines express netrins and their receptors: Endogenous netrin inhibits U87 and U373 cell migration. (A) Western blot analysis of cell lysates or conditioned media from astrocytes (Ast), U343, U373, and U87 cells. Molecular mass markers (kDa) are indicated to the left of each blot. Full-length netrin protein (∼75 kDa) was detected in medium conditioned by each glioma cell line or by astrocytes (top panel). A band corresponding to full-length DCC protein (∼185 kDa) was detected in whole cell lysates of astrocytes and U87 cells, but not U343 and U373 cells. This blot was overexposed to reveal DCC in astrocytes and its absence in U343 and U373 cells. U343 and U373 cells transfected with pDCC-GFP express DCC-GFP chimeric protein, which migrates at a slightly higher molecular weight than endogenous DCC (middle panel). A ∼190 kDa band, the molecular weight of full-length neogenin, was detected in lysates of all three cell lines (bottom panel, 30 µg protein/lane). (B) RT-PCR analysis of U87, U343 and U373 cell total RNA. (C) Transfilter microchemotaxis assays of U87, U343, and U373 motility. § p<0.05 vs. U87. * p<0.05 vs U343. (D) U87 cell migration increased when 25 µg/ml netrin function-blocking antibody (Net_FB_) was added to the top and bottom compartments, relative to medium alone (Control), or control antibody (Control IgG). 10 µg/ml DCC_FB_ did not increase migration. (F) Netrin function-blocking antibody (Net_FB_) significantly increased U373 cell migration, but had no effect on U343 cell migration (E). (G) Schematic diagram of microchemotaxis assay. Number of cells migrated is per 10X objective field. Duration of microchemotaxis assays was 16 hrs. * p<0.05 vs. control.

To determine if netrins regulate glioblastoma cell migration, we first characterized netrin and netrin receptor expression in human astrocytoma cell lines U87, U343, and U373, and in cultures of astrocytes isolated from newborn rat cortex (Fig. 1A). Western blot analysis using an antibody that binds netrin-1 and netrin-3 [20] detected a ∼75 kDa band corresponding to full-length netrin in conditioned medium collected from all cells tested. The DCC_IN_ monoclonal antibody detected a ∼185 kDa band corresponding to DCC in astrocyte and U87 cell lysates. In contrast, DCC was not detected in lysates of U343 or U373 cells. The DCC homologue neogenin was expressed by astrocytes and was detected in all glioblastoma cell lysates.

RT-PCR (Fig. 1B) revealed *dcc* expression by U87 cells but not U343 or U373 cells, and *neogenin* and *unc5* expression by all three cell types. U87 and U343 cells express *unc5b and c*, and U373 cells express *unc5b, c*, and *d*. *Netrin-1* expression was detected in U343 and U373 cells, and *netrin-3* expression in U87 cells. Netrin-1 and netrin-3 are essentially functionally equivalent: both bind DCC and UNC5 proteins and evoke chemoattractant or chemorepellent responses from responsive cells [21].

We then sought to determine if netrin might exert an autocrine influence on cell migration. We first assessed the relative motility of the three cell lines using a transfilter chemotaxis assay as described [22]. Briefly, cells were cultured on the upper surface of a porous membrane (Fig. 1G) and allowed to migrate across. Following migration, cells remaining on the upper surface of the membrane were scraped off, and the cells that migrated to the underside were fixed, stained, and counted. While this assay is often employed to assess the migration of cells in response to a putative attractant or repellent cue, here we used it in the absence of added factors to compare the relative rates of spontaneous migration of the three glioblastoma lines. U343 and U373 cells, lacking DCC, migrated significantly faster than DCC-expressing U87 cells (Fig. 1C). Notably, the U343 cells, which were originally derived from a grade IV glioblastoma multiformae [23], migrated significantly faster than either the U87 or U373 cells, both of which were originally derived from less aggressive grade III astrocytomas [24].

### Autocrine netrin inhibits U87 cell motility

We hypothesized that DCC and netrin expressed by U87 cells might exert a kinetic influence on the rate of cell movement, independent of netrin's influence on directional migration. We therefore tested the effect of disrupting DCC and netrin function on the spontaneous rate of U87 cell migration. The rate of spontaneous migration was not affected by addition of a DCC function-blocking antibody (DCC_FB_). In order to disrupt autocrine netrin function, netrin function-blocking antibody (Net_FB_) was added to both the top and bottom compartments of the migration chamber. This resulted in an approximately 10-fold increase in spontaneous migration across the filter relative to the number of cells migrating in either medium alone (Control) or in the presence of a control IgG (Fig. 1D).

### Autocrine netrin-1 inhibits migration of U373, but not U343, glioblastoma cells

Netrin's capacity to inhibit U87 cell motility in a DCC-independent manner led us to determine if a similar mechanism was active in U343 or U373 cells, which do not express DCC. The addition of netrin function-blocking antibody to both the top and bottom compartments of the transfilter assay significantly increased the rate of U373 migration (Fig. 1F), indicating that endogenous netrin-1 inhibits the rate of U373 migration.

Unlike U87 and U373 cells, blocking netrin function did not alter the rate of U343 cell migration (Fig. 1E). Although U343 cells express neogenin and UNC5 homologue netrin receptors, the absence of an increase in the rate of migration may be the result of mechanisms that restrain inappropriate cell motility being more severely disrupted in these cells.

### Netrin-1 is a chemotropic attractant for U87 glioblastoma cells

**Figure 2:**
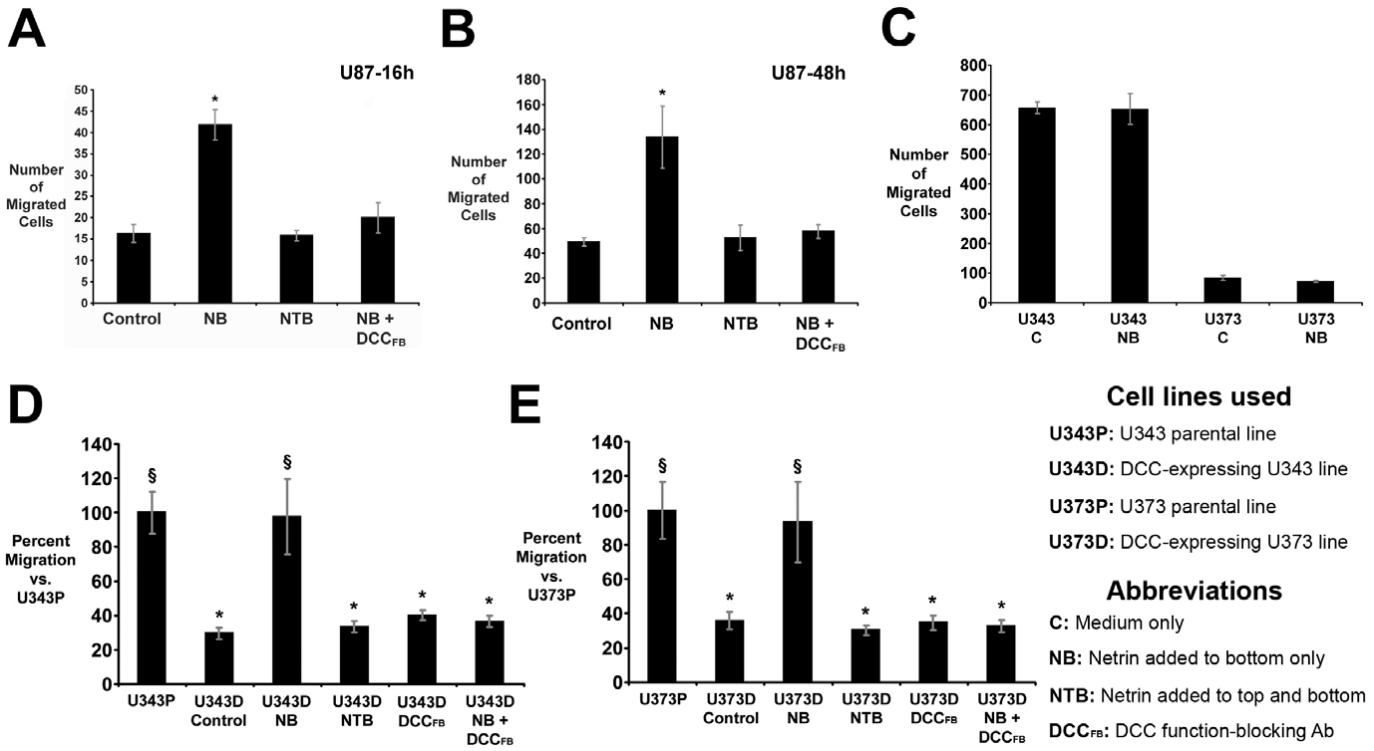
Netrin-1 is a chemoattractant for DCC-expressing glioblastoma cells. (A,B) Addition of 100 ng/ml netrin-1 to the bottom compartment (NB) of the transfilter microchemotaxis assay significantly increased U87 cell migration compared to control (medium alone). NB: netrin-1 bottom; NTB: netrin-1 top and bottom; NB DCC_FB_: netrin-1 bottom, DCC function-blocking antibody top and bottom. Similar results were obtained in assays lasting (A) 16 hours and (B) 48 hours. (C) Netrin-1 in the bottom compartment had no effect on the migration of U343 or U373 cells. (D) U343 cells transfected with a DCC expression construct (U343D control) reduced their rate of migration relative to the parental line (U343P). Increased migration of DCC-transfected U343 cells was evoked by netrin-1 in the bottom compartment (U343D NB), but not uniform netrin-1 (NTB). DCC_FB_ blocked this response (U343D DCC_FB_). (F) Transfection of U373 cells with DCC produced responses similar to U343 cells, which mimic those seen in DCC expressing U87 cells. Number of cells migrated is per 10X objective field. 16 hr assays in all panels except (B). * p<0.05 vs. control (A,B), U343P (D) or U373P (E). § p<0.05 vs. U343D control (E) or U373D control (F).

Transfilter migration assays were then used to determine if DCC-expressing U87 cells can respond to a gradient of netrin-1 as a chemoattractant. Addition of 100 ng/ml netrin-1 to the bottom compartment of the migration chamber (NB) produced a significant increase in the number of U87 cells that migrated across the membrane relative to control (medium alone: Fig. 2A, 16 hr assay; Fig. 2B, 48 hr assay). In contrast, when netrin-1 was added to both the top and bottom compartments (NTB), migration was not significantly different from control. This indicates that U87 cells respond to a gradient of netrin-1 as a chemotropic attractant. When challenged with a gradient of netrin-1 whilst in the presence of the DCC_FB_ antibody in the top and bottom wells (NB DCC_FB_), U87 cell migration was not significantly different from control, indicating that the tropic response of U87 cells to netrin-1 requires DCC. Neither U343 nor U373 cells, which do not express DCC, altered their migration in response to a gradient of netrin-1 (Fig. 2C), despite expressing neogenin and UNC5 netrin receptors. These findings suggest that although these receptors may be sufficient to mediate autocrine inhibition of migration (Fig. 1F), they are insufficient for these cells to generate a chemotropic response to a gradient of netrin-1 (Fig. 2C).

### Chemoattractant response of DCC-expressing U343 and U373 cells to a gradient of netrin-1

To further investigate the contribution of DCC to the regulation of cell motility, we reintroduced the *dcc* gene back into U343 and U373 cells by transfection with a cDNA encoding a DCC-GFP chimera (pDCC-GFP, described by [25]). The proportion of cells expressing DCC-GFP was increased by passaging the cells with Geneticin selection, such that the vast majority of cells seeded in the migration assays expressed DCC. Expression of DCC by U343 and U373 cells was confirmed by western blot (Fig. 1A). Unlike the parental U343 and U373 cell lines, DCC-GFP-expressing U343D and U373D cells migrated up a gradient of netrin-1 (Fig. 2D, E), indicating that ectopic expression of DCC now rendered these cells competent to generate a chemotropic response to netrin-1. Like DCC-expressing U87 cells, the gain-of-function migration towards netrin-1 exhibited characteristics of chemotropic attraction, as the cells only responded to a gradient. Uniform presentation of exogenous netrin-1 resulted in migration that was not significantly different from control. The DCC_FB_ antibody blocked the chemoattractant response of U343D and U373D cells, indicating that DCC is required for chemoattraction to netrin-1.

Consistent with the slow migration of DCC-expressing U87 cells, the number of DCC-transfected U343 and U373 cells that migrated under control conditions was substantially reduced relative to that of the parental cells (Fig. 2D, E). These findings suggest that DCC expression decreases the motility of these cells; however, application of the DCC function-blocking antibody (DCC_FB_) did not increase the rate of migration, as was also found for the U87 cells (Fig. 1D). In constrast, DCC_FB_ completely blocked the chemoattractant migratory response of the U87 cells, and the DCC-transfected U343 and U373 cells to a gradient of netrin-1. These findings are consistent with DCC activating a mechanism that slows non-directional cell migration; however, this mechanism can be differentiated from DCC-dependent chemoattraction due to its insensitivity to DCC_FB_.

### Chemoattraction to netrin-1 is converted to repulsion by laminin-1

**Figure 3:**
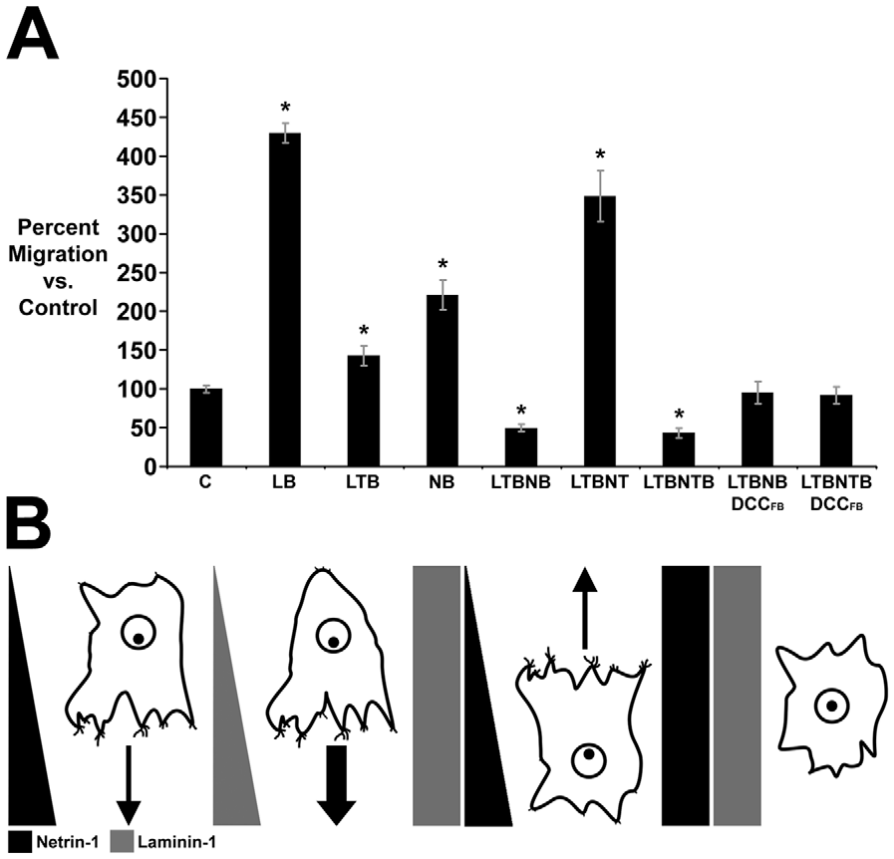
U87 attraction to netrin is converted to repulsion by laminin-1. (A) U87 migration in the microchemotaxis assay challenged with an ascending gradient of laminin-1 (LB) increased relative to control (C). A uniform distribution of laminin-1 (LTB) does not increase U87 migration. An ascending gradient of netrin-1 and uniform laminin-1 (LTB NB), or uniform distributions of both netrin-1 and laminin-1 (LTB NTB), results in reduced U87 migration. Challenging cells with a descending gradient of netrin-1 with a uniform distribution of laminin-1 (LTB NT), evoked increased migration relative to control. Addition of DCC_FB_ to both the top and bottom compartments in the presence of a uniform distribution of laminin-1 and an ascending gradient of netrin-1 (LTB NB DCC_FB_) or of uniform distributions of both netrin-1 and laminin-1 (LTB NTB DCC_FB_) blocked the decrease in migration observed. (B) Schematic depicting migratory responses of U87 cells in (A). Migration assayed after 48 hrs. * p<0.05 vs. control.

Laminin-1 exerts a neuromodulatory influence that converts the response of *Xenopus* retinal ganglion cell growth cones to netrin-1 from attraction to repulsion [26]. We therefore investigated the possibility that laminin-1 might influence the migratory response of U87 cells to a gradient of netrin-1 (Fig. 3). When U87 cells were challenged with an ascending gradient of laminin-1 (LB), the number of cells that migrated across the membrane increased. In the presence of a uniform concentration of laminin (LTB), U87 migration was not significantly different from control, indicating that a gradient of laminin-1, like netrin-1, is a chemoattractant for these cells. Interestingly, the combination of an ascending gradient of netrin-1 and a uniform concentration of laminin-1 (LTB NB) dramatically reduced the number of U87 cells that migrated across the membrane to the extent that it was significantly less than control, suggesting that laminin-1 converted the reponse to netrin-1 from attraction to repulsion. Consistent with this, confronting cells with a descending netrin-1 gradient in the presence of a uniform concentration of laminin-1 (LTB NT) resulted in an increase in migration relative to control. Importantly, this finding provides strong evidence that laminin-1 does not influence the response to a gradient of netrin-1 by arresting cell motility. When the cells were simultaneously exposed to uniform concentrations of netrin-1 and laminin-1, (LTB NTB), fewer cells migrated across the membrane, indicating that the combined action of netrin-1 and laminin-1 exerts a non-directional effect that inhibits U87 cell motility. These results are consistent with laminin-1 switching netrin-1 from an attractant to a repellent for U87 cells, as previously described for the axons of *Xenopus* retinal ganglion cells [26]. Addition of DCC_FB_ antibody in the presence of a uniform concentration of laminin-1 and either an increasing gradient (LTB NB DCC_FB_) or uniform concentration (LTB NTB DCC_FB_) of netrin-1, did not significantly affect migration compared to control, indicating that the laminin-induced repellent response to netrin-1 requires DCC. This is consistent with a requirement for DCC in chemorepellent responses to netrin-1, documented in many cell types [27] [28], including glial precursor cells [29]–[31] [22] [32].

### Netrin-1 and DCC do not affect U87, U343, and U373 cell proliferation or survival

**Figure 4:**
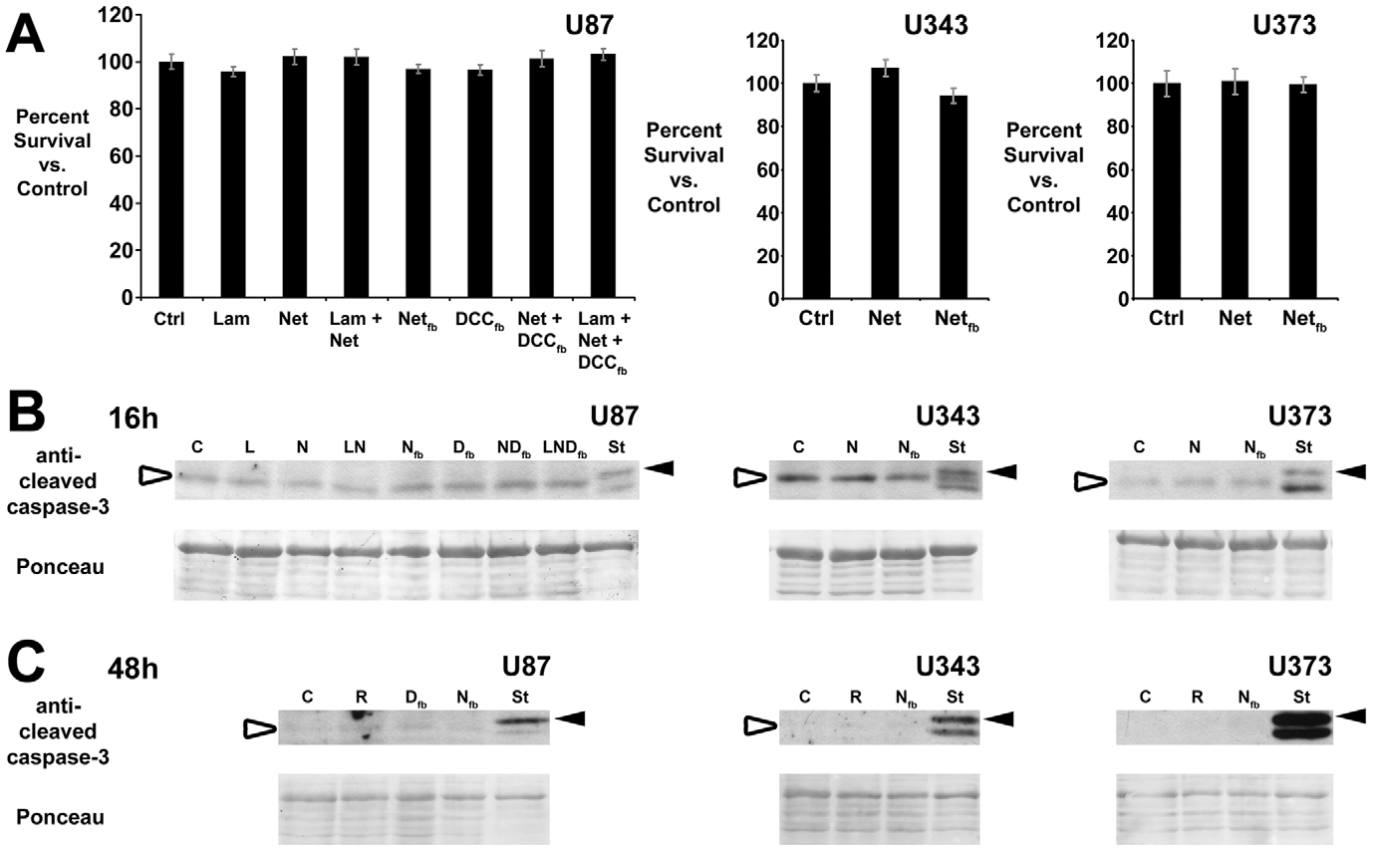
Neither netrin-1 nor laminin-1 affect the survival or proliferation of U87, U343, or U373 cells. (A) Cell viability was assessed by labeling F-actin with Alexa 488-conjugated phalloidin, nuclei with Hoechst, and counting the number of cells. Addition of netrin-1, laminin-1, or both did not affect U87 cell viability. Neither 25 µg/ml Net_FB_ nor 10 µg/ml DCC_FB_ affected cell number. The number of U343 or U373 cells did not change following addition of 100 ng/ml netrin-1 or 25 µg/ml Net_FB_ (16 hr assay). (B) To further assess apoptotic cell death under the same conditions analyzed in panel A, cell lysates were analyzed by immunoblot for the active (cleaved) form of caspase-3. In all three cell lines, a 17 kDa caspase-3 band (black arrowhead) was only observed in lysates exposed to staurosporine, a potent inducer of apoptosis. The white arrowhead indicates a nonspecific 15 kDa immunoreactive band. (C) To determine if netrin regulates apoptosis through a ‘dependence’ mechanism, cells were treated with antibodies blocking either DCC or netrin function for 48 hours. As in panel B, only staurosporine treatment promoted cell death. Ponceau S staining demonstrates equal loading. Ctrl C control; Lam L laminin-1; Net N netrin-1; Net_fb_ N_fb_ netrin function-blocking antibody; DCC_fb_ D_fb_ DCC function-blocking antibody; LN laminin-1 and netrin-1; ND_fb_ Netrin-1 and DCC_fb_; LND_fb_ Laminin-1 netrin-1 and DCC_fb_; R pre-immune rabbit IgG; St staurosporine.

DCC and UNC5 homologues have been proposed to function as dependence receptors, activating apoptosis in the absence of netrin-1 [33]. This raises the possibility that the effects described above may be due to an influence on cell survival and not motility. Thus, we examined the consequences of manipulating netrin function on the survival of U87, U343 and U373 cells. No significant change in cell number (Fig. 4A), or activation of caspase-3, an indicator of apoptosis (Fig. 4B), was detected following 16 hrs treatment with exogenous netrin-1, laminin-1, or both; nor following disruption of netrin or DCC function using blocking antibodies. Further testing, by blocking netrin and DCC function for 48 hrs, again resulted in no detectable increase in caspase-3 activation (Fig. 4C). In contrast, staurosporine, applied as a positive control, activated caspase-3 and caused extensive cell death (Fig. 4B, C). These findings are consistent with previous analyses of glial precursor cells, indicating that netrin-1 and DCC do not regulate oligodendroctye precursor survival either *in vitro* or *in vivo* [22] [34], and they support the conclusion that the results of transfilter assays reflect changes in cell migration and not effects on cell survival or proliferation.

### Endogenous netrin promotes the maturation of focal complexes into focal adhesions

Cell migration requires the formation of transient adhesive contacts with the extracellular matrix (ECM). Initial contacts occur at the leading edge of lamellipodia where integrins bind ECM ligands and recruit proteins such as vinculin and paxillin to form immature adhesive contacts called focal complexes (FC) (reviewed by [35]). The transition from FC to FA is marked by consolidation of the adhesive contact, an increase in size, and the recruitment of additional proteins including zyxin [36].

**Figure 5:**
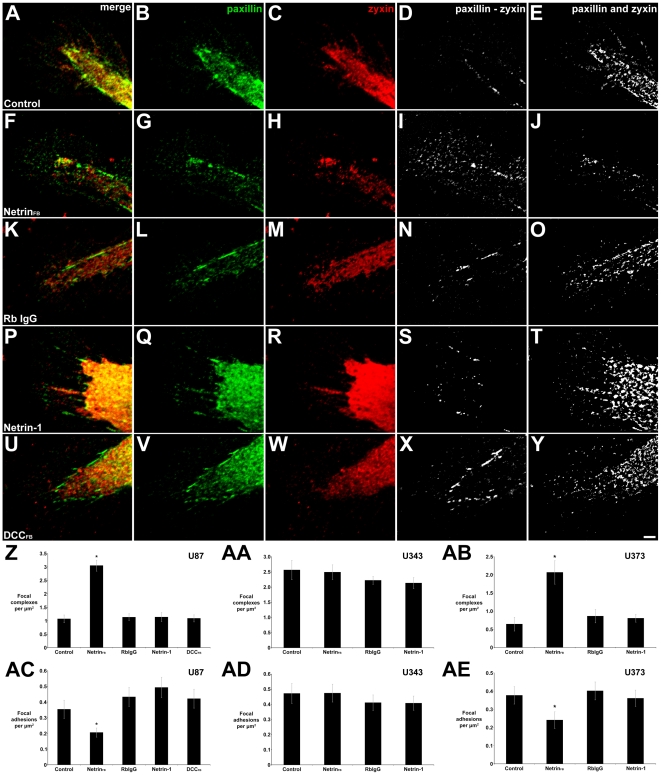
Disrupting netrin function increases the number of FCs and reduces the number of FAs in lamellipodial protrusions of U87 and U373, but not U343, cells. (A, F, K, P, U) U87 cells were labeled with antibodies against paxillin (green) and zyxin (red). FCs present in lamellipodia of U87 cells were identified and quantified by subtracting zyxin immunoreactivity (C, H, M, R, W) from paxillin immunoreactivity (B, G, L, Q, V), revealing localization of paxillin without zyxin (D, I, N, S, X). (Z) Density of paxillin+/zyxin- foci. FAs in U87 cell lamellipodia were identified and quantified by generating images of paxillin and zyxin co-localization (E, J, O, T, Y) and determining the density of paxillin+/zyxin+ foci (AC). 25 µg/ml control rabbit IgG (Rb IgG; K–O), 100 ng/ml netrin-1 (P–T) or 10 µg/ml DCC_FB_ (U–Y) resulted in no change in FC or FA density relative to control medium (A–E). 25 µg/ml Net_FB_ (F–J) significantly increased the density of FCs (Z) and decreased FA density (AC). FCs and FAs of U373 cells were similarly affected (AB, AE). FC of FA density was not altered by control antibody, netrin-1, or Net_FB_ in U343 cells (AA and AD). 100x objective, scale bar  = 2 µm. * p<0.05 vs. control.

The effect of disrupting netrin function on adhesive complex formation in glioblastoma cells was investigated by examining the distribution of paxillin, which is present in both FAs and FCs, and zyxin, which is present in FAs but not FCs. The influence of netrin on FC formation was quantified by subtracting the distribution of zyxin immunoreactivity (Fig. 5C, H, M, R, W) from paxillin immunoreactivity (Fig. 5B, G, L, Q ,V) to create images representing regions of paxillin, but not zyxin localization (Fig. 5D, I, N, S, X). Using the ‘paxillin minus zyxin’ images, the density of FCs present in each lamellipodium was calculated. Exposure of U87 cells to an isotype control antibody (RbIgG), DCC_FB_, or netrin-1, resulted in no change relative to control. In contrast, application of Net_FB_ resulted in increased FC density (Fig. 5Z). A similar increase was observed when netrin function was inhibited in U373 cells, but not U343 cells, in which FC density was high in all conditions examined (Fig. 5AA and AB, data not shown).

To determine if inhibiting endogenous netrin function influences FA density, images depicting regions of paxillin and zyxin colocalization were generated (Fig. 5E, J, O, T, Y). From the ‘paxillin and zyxin’ images, the density of FAs in each lamellipodium was calculated. In U87 (Fig. 5AC) and U373 (Fig. 5AE) cells, addition of netrin function-blocking antibody resulted in decreased FA density. In all other conditions analyzed for U87 and U373 cells and in all conditions analyzed for U343 cells, no change in FA density was observed.

Notably, the increase in FC density and corresponding decrease in FAs correlates precisely with the changes in motility evoked by disrupting netrin function and measured using the microchemotaxis assay (Fig. 1). The absence of an effect of the DCC function-blocking antibody provides evidence that DCC function is not essential for this non-directional effect of netrin on motility, consistent with the lack of an effect of disrupting DCC function on the rate of cell migration (Fig. 1). That the addition of exogenous netrin-1 protein did not influence FC or FA density suggests that the relatively high level of netrin protein secreted by the cells is sufficient to saturate the inhibitory response in the conditions described here. These data are consistent with a mechanism in which autocrine secretion of netrin promotes the maturation of FCs into FAs, and that the accumulation of these adhesive structures restrains cell movement.

### Netrin and netrin receptors are localized to focal adhesions, but not focal complexes

**Figure 6:**
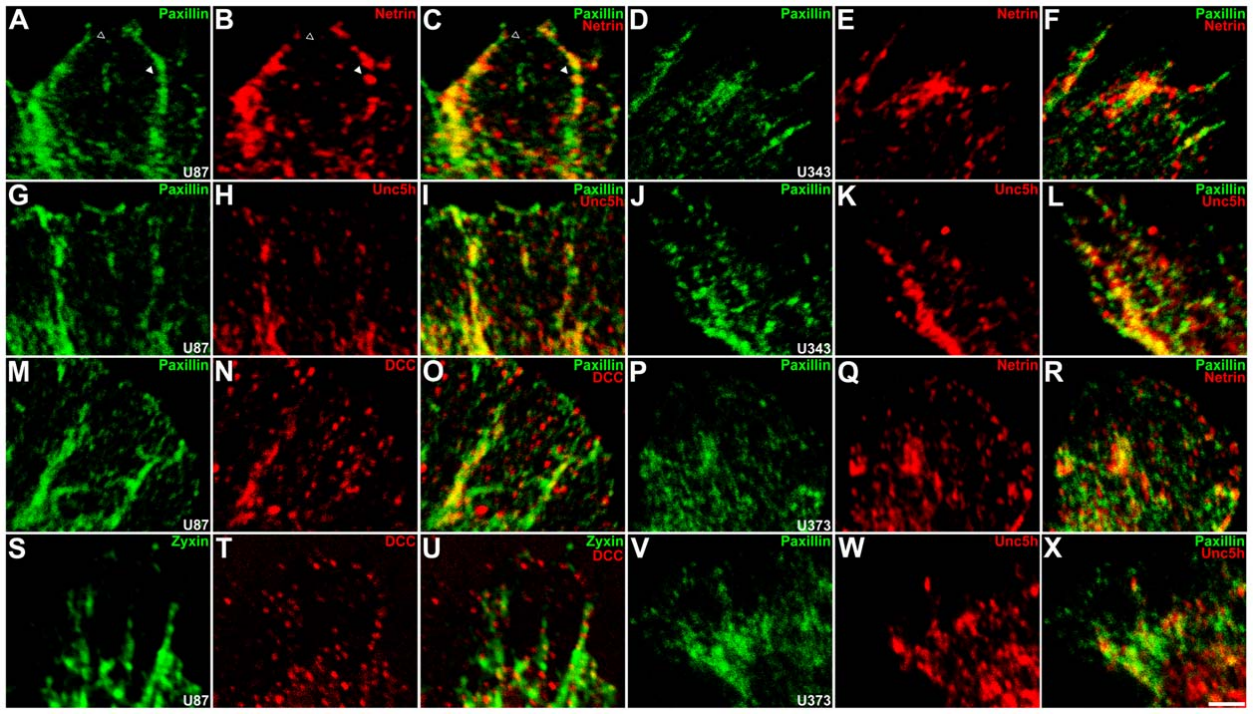
Netrin and netrin receptors are localized to FAs but not FCs. U87, U343, and U373 cells were labeled with antibodies against paxillin (green) and netrin, DCC, and UNC5 proteins (red; all panels except S–U) or zyxin (green) and DCC (red; S–U), and lamellipodia imaged. In U87 cells, small, paxillin-positive FCs localized at the lamellipodial edge were not netrin-positive (black arrowhead). Netrin immunoreactivity co-localizes with larger paxillin-positive structures located away from the lamellipodial edge (white arrowhead), consistent with FAs (A–C). UNC5 (G–I) and DCC (M–O) immunoreactivity were similarly localized to FAs in U87 cells. DCC immunoreactivity also co-localized with zyxin-positive FAs (S–U). Similarly, in U343 and U373 cells, netrin (D–F, P–R) and UNC5 proteins (J–L, V–X) co-localize with FAs, but not FCs. 100x objective, scale bar  = 2 µm.

We next investigated the possibility that netrin and netrin receptors might be localized to FCs or FAs and thereby directly influence their maturation. U87, U343, and U373 cells were labeled with antibodies against paxillin and one of DCC, netrin, or UNC5 proteins. U87 cells were also labeled with anti-DCC and anti-zyxin (Fig. 6). In U87 cells netrin (Fig. 6A–C), DCC (Fig. 6M–O) and UNC5 (Fig. 6G–I) immunoreactivity colocalized with large paxillin-positive foci characteristic of FAs (white arrowhead), but not smaller paxillin-positive structures characteristic of FCs (black arrowhead). In U343 and U373 cells that lack DCC expression, netrin (Fig. 6D–F, P–R) and UNC5 (Fig. 6J–L, V–X) immunoreactivity was similarly localized to FAs but not FCs. Consistent with localization to FAs, DCC and zyxin immunoreactivity colocalized in U87 cells (Fig. 6S–U). Colocalization with markers of FAs is consistent with netrins and netrin receptors regulating cell-substrate adhesion and motility.

## Discussion

Here we provide evidence that secreted netrins can function as autocrine inhibitors of cell motility. Our findings support the conclusion that DCC is required for cells to migrate directionally in response to a gradient of netrin-1. Ectopic DCC expression conferred on U343 and U373 cells the capacity to respond to a gradient of netrin-1. DCC expression also slowed the rate of spontaneous migration in these cells, consistent with DCC restraining cell movement. The glioblastoma-derived cell lines tested express either *netrin-1* (U343, U373) or *netrin-3* (U87). Disrupting endogenous netrin-1 or netrin-3 function dramatically increased the rate of U87 and U373 cell movement. U87 cells express DCC while U373 cells do not, indicating that in addition to DCC slowing cell migration, netrins must influence the motility of these cells through a DCC-independent mechanism. Unc5 homologue netrin receptors are required for axonal growth cone repulsion and collapse induced by netrin-1, and co-expression of DCC often facilitates UNC5 function (reviewed by [1]). Our findings support the hypothesis that UNC5 proteins, in collaboration with DCC, underlie the netrin-mediated inhibition of motility described here; however the role of UNC5 homologues in these cells remains to be tested directly.

Consistent with increasing the rate of cell motility, disrupting endogenous netrin function increased the number of lammelipodial FCs, immature adhesive contacts that are associated with cell movement. Netrin, DCC, and UNC5 immunoreactivity was co-localized with FA but not FC markers, suggesting that netrin may act at the nascent FA itself to promote the maturation of FCs to FAs.

### Netrin, focal adhesions, and cell motility

Netrin-1 signaling through DCC directs the organization of F-actin [25], regulating the activation of RhoGTPases, PAK1, MAPK, FAK, and Src family kinases [25] [37]–[44]. FAK and Src are also activated downstream of UNC5 proteins in response to netrin [45] [46]. FAs are sites of interaction for many proteins [35]. Our evidence indicates that netrin and netrin receptors are localized to FAs. We hypothesize that netrins may contribute to restricting cell movement by promoting FA maturation. Numerous proteins present in FAs have been implicated in signaling downstream of netrin: FAK, Src, the Ena/VASP proteins [47], Rho-family GTPases Cdc42, Rac, and RhoA [25] [48] [43] and the GEF Trio [49]. FAK is activated by autophosphorylation that creates a binding site for Src-family kinases. Association with FAK initiates a FAK-Src signaling complex. Extensive tyrosine phosphorylation is a key signaling event observed in focal adhesions, as it is thought to create ‘docking’ sites for recruitment of SH2 domain-containing proteins required for further signaling events (reviewed by [50]). FAK and Src regulate the phosphorylation of UNC5B on multiple tyrosine residues upon netrin binding, and that following these phosphorylation events, Src associates directly with UNC5B via its SH2 domain. Interestingly, this is enhanced by, but does not absolutely require, DCC function [46], perhaps reflecting that co-expression of DCC can facilitate UNC5 function (reviewed by [51]). Notably, FAK is required for the maturation of adhesive complexes [52], and, together with Src, is essential for the normal turnover of FAs (reviewed by [50]). The findings we present here provide a foundation for investigating the role of netrin-1 in the formation of focal adhesions.

### An emerging role for netrin in adhesion and tissue morphogenesis

Netrin-1 and netrin-3 are secreted proteins, which raises the question of how they may contribute to anchoring a cell to either the substrate or another cell. The majority of netrin-1 protein in the CNS is not freely diffusible, but is bound to cell surfaces and extracellular matrix [5] [53](reviewed by [51]). DCC binding immobilized netrin-1 mediates cell-substrate adhesion, consistent with a role for netrin mediating cell-matrix interactions [38] [48]. Key roles for netrins and netrin receptors have been identified during tissue morphogenesis (reviewed by [54]), including development of the mammary epithelium [55], pancreas [56], lung [57], lymphangiogenesis [58], and during angiogenesis [59] [60]. Furthermore, overexpression of netrin-1 by cells in the intestinal epithelium of mice led to the formation of focal hyperplasias and adenomas [61]. These authors concluded that the phenotype induced was due to netrin-1 reducing cell death; however, our findings raise the possibility that disruption of appropriate cell-cell interactions as a result of netrin-1 overexpression may contribute to the disorganization of normal epithelial structure.

### How might secreted netrins influence tumor cell migration in vivo?

Our findings suggest that loss of netrin function may lead to disruption of appropriate cell-cell and cell-matrix interactions. We have provided evidence that in the presence of laminin-1, netrin-1 becomes a repellent for U87 cell migration, and that this requires DCC (Fig. 3). Importantly, the combined action of netrin-1 and laminin-1 may influence glioblastoma cell migration *in vivo*. Laminin-1 is restricted to basement membranes and capillary walls in developing and mature CNS [62]–[64]. Although deregulated cell migration makes an important contribution to the dissemination of tumor cells within the brain, metastasis of brain tumor cells outside the CNS is rare. Glioma cells are attracted to endothelial capillaries *in vitro* [65] and glioblastoma cells migrate in close association with capillary walls as they disseminate within the brain [66]. Laminin-1 may facilitate this as it promotes glioma cell migration [67] [68]. Based on our evidence that laminin-1 biases cells to respond to netrin-1 as a repellent (Fig. 3, see also [26]), the basal lamina may inhibit the migration of glioma cells expressing netrin-1 and DCC. In contrast, in the absence of netrin function, our findings predict that deregulation of this inhibition of migration will lead to laminin-1 in the basal lamina of blood vessels becoming a permissive substrate that promotes tumor cell migration and dissemination to other brain regions. Correlated loss of DCC expression with tumor progression suggests that netrin and DCC may play an important role in tissue maintenance in adulthood. We propose that appropriate cellular organization may be stabilized by autocrine and paracrine actions of netrin. Our findings suggest that loss of effective netrin signaling may disinhibit a mechanism that normally restrains cell migration. In the absence of netrin-mediated inhibition, local cues such as laminin, are predicted to become potent promoters of migration.

Numerous cell types expressing both netrin and netrin receptors *in vivo* have been described. We provide evidence that autocrine expression of netrin can restrain cell migration, and promote the maturation of focal complexes into focal adhesions. These findings identify a novel netrin function that may contribute to the formation and maintenance of tissue organization, and identify netrin and its receptors as potential therapeutic targets to inhibit tumor cell migration and dispersion.

## Materials and Methods

### Cells and cell culture

Human glioblastoma cell lines, U87, U343, U373 (ATCC, Rockville, MD) and astrocytes isolated from newborn mouse brain were grown as monolayer cultures in DMEM (Invitrogen, Burlington, ON), 10% heat-inactivated fetal bovine serum (FBS, Invitrogen), 1% glutamax-1 (Invitrogen) and 1% penicillin/streptomycin. All procedures using animals were performed in accordance with the Canadian Council on Animal Care guidelines for the use of animals in research, and were approved by the animal care review board of the Montreal Neurological Institute (approval ID # 4330).

### Antibodies, conditioned media, cell lysates, western blotting, and PCR

Antibodies against the following were used: cleaved caspase-3 (Asp175, mouse, Cell Signaling Technology, Beverly, MA); DCC (DCC_IN_, mouse, G97-449; BD Biosciences Pharmingen, San Jose, CA; DCC_GT_, goat, A-20; Santa Cruz Biotechnology, Santa Cruz, CA; function-blocking, DCC_FB_, mouse, AF5; Calbiochem, La Jolla, CA); netrin-1 and 3 (PN2, rabbit [5]); netrin function-blocking (Net_FB_, PN3, rabbit [5]); neogenin (rabbit, Santa Cruz Biotechnology); paxillin (mouse, BD Biosciences Pharmingen); Unc5C (rabbit [45], provided by Dr. Tony Pawson, Mount Sinai Hospital, Toronto, ON; isotype control rabbit IgGs (RbIgG; Invitrogen); and zyxin (rabbit, Abcam, Cambridge, MA).

Cultures were grown to 80% confluence and conditioned media collected following 48 hrs in serum-free DMEM. For lysates, cells were grown to 80% confluence, rinsed with PBS and lysed in 1 ml of hot sample buffer (60 mM Tris/HCl, pH 6.8, 2% SDS, 10% glycerol, 100 mM DTT). For western blot analysis of cleaved caspase-3, cells were cultured at a density of 120,000 cells/well in a 12-well tissue culture plate. Nitrocellulose immunoblots were probed with DCC_IN_ (0.5 µg/ml), PN2 anti-netrin (4 µg/ml), anti-cleaved caspase-3 (1∶1000), or anti-neogenin (0.4 µg/ml). After washing, membranes were incubated with HRP-coupled secondary antibodies and immunoreactivity visualized using chemiluminescence (NEN, MA).

PCR was carried out using standard methods. Total RNA was isolated from glioblastoma cells using Trizol (Life Technologies, MD) and cDNA prepared using SuperScript II reverse transcriptase (Invitrogen). Primers were annealed at 60°C to amplify *netrin-1* (527 bp), *netrin-3* (429 bp) and *dcc* (434 bp), 58°C for *neogenin* (545 bp) and *unc5C* (530 bp), and 55°C for *unc5A* (215 bp), *unc5B* (350 bp), and *unc5D* (324 bp).

human *netrin-1* F: 5′GCCGCCACTGCCATTACTGC 3′

R: 5′GAGGGGCTTGATTTTGGGACACTT 3′,

human *netrin-3* F: 5′CCGCTGGGCTTCTTCTCC 3′

R: 5′GCAGCGGCCGCAGTCAGG3′,

human *dcc* F: 5′CAAGTGCCCCGCCTCAGAACG 3′

R: 5′GCTCCCAACGCCATAACCGATAAT 3′,

human *neogenin* F: 5′ TGGCCCAGCACCTAACCT 3′

R: 5′TTGCCGGGCCTGTACCATTGATTG 3′

human *unc5a* F: 5′ TCGTCAAGAACAAGCCAGTG 3′

R: 5′ GCACTGGCACCAGTATTC 3′

human *unc5b* F: 5′ TCCAGCTGCATACCACTCTG 3′

R: 5′ AGCCACCAGCATCTCACTCT 3′

human *unc5c* F: 5′ GCCAGCAAGTGGAAGAACTC 3′

R: 5′ CACACTCTGCCCTTCACAGA 3′

human *unc5d* F: 5′ ATATTCCCCCATTCCTCTGG 3′

R: 5′ TAGCACAAATCCGCTGTCGTCTG 3′

### Transfilter chemotaxis assay

Cells were plated at a density of 4×10^5^ cells/ml on polycarbonate transwell culture inserts (6.5 mm diameter with 8 µm pore size, Corning). 100 µl of cell suspension was applied to the upper surface of the filter, and the filters placed in the wells of a 24-well plate over 600 µl of medium. DMEM with 0.2% BSA, 100 U/ml penicillin, 100 µg/ml streptomycin, and 2 mM glutamax was the base medium used for all assay conditions. Following migration, cells on the upper side of the filter were scraped off, and the cells attached to the lower side of the filter fixed with 4% paraformaldehyde (PFA)/0.1% glutaraldehyde (30 min, 4°C). Filters were rinsed with PBS, and cell nuclei stained with Hoechst dye. Four transwells were used per condition. Four images of each filter were captured using a 10 X objective and nuclei counted using Northern Eclipse software (Empix Imaging, TO). Where pooled results are presented, the value ‘percent migration vs. control' for a given trial represents the number of cells migrated in that condition expressed as a percentage of the mean number of cells migrating in control conditions. Recombinant netrin-1 protein was purified as described [38] and used at a concentration of 100 ng/ml. Laminin-1 was used at 10 µg/ml (BD Biosciences, Bedford, MA). Net_FB_ and rabbit isotype control IgG (used as a control) were added at a concentration of 25 µg/ml. DCC_FB_ was added at a concentration of 10 µg/ml. Statistical significance was calculated using Student's t-test and error bars represent S.E.M.

### Plasmids and transfection

U343 and U373 cells were transfected using lipofectamine (Invitrogen) with expression constructs encoding either green fluorescent protein (GFP) alone or DCC tagged at its C-terminus with GFP [25]. Seventy-two hrs after transfection, the medium was changed to selection medium containing Geneticin (Invitrogen).

### Confocal image analysis

10^4^ cells were plated per well in chamber slides (Fisher) coated with 20 µg/ml poly-D-lysine (Sigma) at 4°C overnight, washed with Hanks buffered salt solution (Invitrogen) and allowed to dry. Cells were fixed in 4% PFA, 4% sucrose in PBS, and permeabilized with 0.25% Triton X-100 in PBS. Blocking was performed in 3% heat-inactivated normal goat serum, 2% BSA, and 0.125% Triton X-100 in PBS. Cells were then incubated with anti-paxillin and anti-zyxin (Fig. 5), anti-paxillin and one of anti-netrin PN2, anti-UNC5, or anti-DCC_GT_, or anti-zyxin and anti-DCC_GT_ (Fig. 6) diluted in blocking solution. Primary antibodies were detected with secondary antibodies coupled to Alexa 546 or Alexa 488 (Molecular Probes).

For imaging adhesive complexes, single confocal optical slices through the base of lamellipodia were collected. Identical settings were used for each condition examined for a given cell line. The outermost region of individual lamellipodial protrusions (excluding regions of paxillin or zyxin immunoreactivity contiguous with adhesive structures in the cell body) was outlined using Image J software [69]. Mask images were then generated representing either the regions staining with both paxillin and zyxin (using the ‘AND’ function) or representing the difference between the paxillin and zyxin images (the zyxin signal subtracted from the paxillin image). Images were adjusted to eliminate signal below a minimum value that was held constant across all images for each cell line. Signal in the ‘AND’ image corresponds to the area of each lamellipodium occupied by mature FAs that contain both paxillin and zyxin. To quantify FCs, the subtracted image representing paxillin staining without zyxin was filtered to exclude structures smaller than 3 pixels or larger than 40 pixels. The number of individual adhesions was then counted and the density of adhesions within each lamellipodium calculated. Netrin-1 was used at 100 ng/ml, Net_FB_ and rabbit preimmune IgG (as a control) at 25 µg/ml, and DCC_FB_ at 10 µg/ml.

### Analysis of cell number and apoptosis

To investigate changes in cell survival or proliferation, cells were plated at a density of 30,000 cells per well in 8-well chamber slides (Fisher), allowed to settle for 2 hrs, treated as described for 16 hrs, fixed and stained with Alexa-488 conjugated phalloidin and Hoechst, and the number of live cells per 20 X field counted. To measure levels of apoptosis in these cultures, 120,000 cells were cultured in each well of a 12-well tissue culture plate, allowed to settle for 2 hrs, and treated as described for either 16 or 48 hrs. In all cases, the base medium used was DMEM with 2% FBS, penicillin/streptomycin, and glutamax-1. Statistical significance was calculated using Student's t-test and error bars represent S.E.M.
